# G6PD-NF-κB-HGF Signal in Gastric Cancer-Associated Mesenchymal Stem Cells Promotes the Proliferation and Metastasis of Gastric Cancer Cells by Upregulating the Expression of HK2

**DOI:** 10.3389/fonc.2021.648706

**Published:** 2021-02-26

**Authors:** Bin Chen, Tuo Cai, Chao Huang, Xueyan Zang, Li Sun, Shuwei Guo, Qianqian Wang, Zhihong Chen, Yuanyuan Zhao, Zhiqiang Han, Rongman Xu, Wenrong Xu, Mei Wang, Bo Shen, Wei Zhu

**Affiliations:** ^1^School of Medicine, Jiangsu University, Zhenjiang, China; ^2^Department of Surgery, Zhenjiang First People's Hospital, Zhenjiang, China; ^3^Department of Orthopedics, Affiliated Hospital of Jiangsu University, Zhenjiang, China; ^4^Department of Clinical Laboratory, Haian People's Hospital, Haian, China; ^5^Department of Oncology, Jiangsu Cancer Hospital Affiliated to Nanjing Medical University, Nanjing, China

**Keywords:** gastric cancer mesenchymal stem cell, gastric cancer, G6PD, HGF, HK2

## Abstract

**Background:** Tumor-associated stromal cells have been widely recognized for their tumor-promoting capability involving paracrine signaling. However, the underlying mechanism and the effects of the molecules in the glycolysis pathway in gastric cancer-associated mesenchymal stem cells (GCMSCs) and gastric cancer cells on tumor progression remain unclear.

**Methods:** The expression of hepatocyte growth factor (HGF) in GCMSCs and bone marrow mesenchymal stem cells (BMMSCs) was detected by enzyme-linked immunosorbent assay (ELISA). The effect of HGF derived from GCMSCs on the proliferation, metastasis, and HK2 expression of gastric cancer cells was evaluated *in vitro* and *in vivo*. The effects of G6PD on the production of HGF in mesenchymal stem cells (MSCs) were analyzed by immunoblotting.

**Results:** HGF derived from GCMSCs promoted glycolysis, proliferation, and metastasis of gastric cancer by upregulating c-Myc-HK2 signal. The progression of the disease induced by GCMSCs decelerated in the absence of HK2. The expression of G6PD activated NF-κB signaling and stimulated the production of HGF in GCMSCs. Blocking HGF derived from GCMSCs decreased proliferation, metastasis, and angiogenesis of gastric cancer cells *in vivo*.

**Conclusions:** GCMSCs highly expressed G6PD and facilitated the progression of gastric cancer through the G6PD-NF-κB-HGF axis coordinates. Blocking HGF derived from GCMSCs is a potential new therapeutic target for the treatment of gastric cancer.

## Introduction

Gastric carcinoma, which is a type of digestive system tumors, is associated with high morbidity and mortality (1). In ischemic and hypoxic tumor microenvironments, the Warburg effect promotes the tumor cells to make efficient use of cellular nutrients (2). Moreover, tumor cells interact with stromal cells to facilitate disease progression (3). Due to the importance of the Warburg effect on tumor progression, further understanding of the biology of the stromal and tumor cells is essential.

Previous work, including our own, has demonstrated that tumor mesenchymal stem cells (MSCs), which belong to stromal cells in the tumor microenvironment, can promote tumor development through paracrine signaling (3–6). MSCs differ from gastric cancer mesenchymal stem cells (GCMSCs) in cytokine production (7). Glucose-6-phosphate dehydrogenase (G6PD) and hexokinase 2 (HK2) are key enzymes in glucose metabolism and play important roles in regulating tumor growth, metastasis, apoptosis, vasculature, and autophagy (8–14). It has been reported that overexpression of G6PD promotes the activation of NF-κB, which can regulate the production of various cytokines (15–17). As G6PD is highly expressed in gastric cancer tissues, it is speculated that it plays a key role in regulating cytokine production.

Accumulating evidence has shown that elevated levels of HK2 lead to various malignant behaviors of cancer cells. HK2 binds to the outer mitochondrial membrane via interactions with the voltage-dependent anion channel (VDAC). It has been determined that mitochondria-bound HK2 not only promotes glycolysis, but also inhibits apoptosis by maintaining the mitochondrial membrane potential and preventing the release of cytochrome C (18, 19). Similarly to other kinases, the transcription and post-translational modification of HK2 is regulated by a variety of factors, including insulin, HIF-1α, c-Src, and c-Myc (20–23). It has been reported that MSCs promote the aerobic glycolysis of leukemic cells by affecting uncoupling protein 2 or activating the AKT-mTOR signaling (24, 25). On the other hand, bone marrow mesenchymal stem cells (BMMSCs) promote glycolysis of gastric cancer cells by upregulating c-Myc (26). Compared with BMMSCs, GCMSCs produce more cytokines, which play important roles in regulating c-Myc (7). Several studies indicate that GCMSCs have the potential to facilitate glycolysis by regulating HK2. Despite hypoxia being a common activator of tumor glycolysis, the role of GCMSCs in tumor glycolysis remains elusive.

In the present study, we found that G6PD-NF-κB signaling in GCMSCs facilitated the production of HGF, which could activate the MET receptor. Furthermore, we demonstrated that HGF derived from GCMSCs promoted the proliferation and metastasis of gastric cancer cells by upregulating HK2. The results presented herein demonstrated the important role of GCMSCs in the glycolysis of gastric cancer cells and provided a theoretical basis for the development of new therapies for gastric cancer.

## Materials and Methods

### *In vivo* Study in a Gastric Cancer Cell Line Xenograft Model

A xenograft tumor model was used to investigate the effects of GCMSCs on the proliferation and metastasis of gastric cancer cells. A total of 35 BALB/c-nu/nu male mice (aged 4–6 weeks) were purchased from Nanjing Biomedical Research Institute of Nanjing University (Nanjing, China). The study complied with the guidelines of the National Institute of Health regarding the care and use of laboratory animals (NIH Publication No. 8023, revised 1978). The procedures were approved by the local ethics committee of the Jiangsu University, China. The animals were raised in the Laboratory Animal Center of Jiangsu University. The mice were maintained in pathogen-free conditions under 12 h light/12 h dark cycle with sterilized feed and autoclaved water. For the tumor proliferation experiment, 20 mice were subcutaneously injected in the flank with 1 × 10^6^ MGC-803 cells in 200 μL PBS. Seven days later, the mice were randomly assigned to four groups (*n* = 5 mice per group). The mice in the control, GCMSC-CM, GCMSC-CM+IgG, and GCMSC-CM+anti-HGF groups were injected every 2 days with 200 μL of DMEM, GCMSC-CM, GCMSC-CM+IgG (300 ng/mL), and GCMSC-CM+anti-HGF (300 ng/mL) at the peri-tumor tissue, respectively. Tumor sizes were measured every 2 days using Vernier calipers. Moreover, tumor volumes were calculated using the following formula: tumor volume = length × width^2^/2. The experiment was ended at 28 days after first injection. All mice were euthanized by carbon dioxide asphyxiation. The tumors were subsequently resected and weighed. For the tumor metastasis experiment, 15 mice were abdominally injected with 2 × 10^6^ HGC-27 cells in 200 μL PBS. After 7 days, the mice were randomly assigned to three groups (*n* = 5 mice per group). The mice in the control, GCMSC-CM, and GCMSC-CM+anti-HGF groups were abdominally injected every 2 days with 200 μL of DMEM, GCMSC-CM, and GCMSC-CM+anti-HGF (300 ng/mL), respectively. The experiment was ended at 28 days after first injection. All mice were euthanized by carbon dioxide asphyxiation. The tumors were subsequently resected and weighed.

### Cell Culture

Five cases of gastric cancer tissues were collected in the Affiliated Hospital of Jiangsu University and informed consents were obtained from all patients. All protocols were approved by the local ethics committee of the Affiliated Hospital of Jiangsu University, China. Isolation and identification of GCMSCs were performed according to a previously described method (27). Briefly, fresh gastric cancer tissues were cut into small pieces of 1 mm^3^. The tissue pieces were attached to petri dishes and cultured with 10% DMEM under sterile conditions at 37°C in a humidified incubator infused with 5% CO_2_. Adherent cells were collected and maintained in DMEM supplemented with 10% FBS at 37°C in 5% CO_2_. Bone marrow cells were collected in the Department of Orthopedics at the Affiliated Hospital of Jiangsu University. Informed consents were obtained from all patients. Three cases of BMMSCs from patients with bone trauma were used as controls. All protocols were approved by the local ethics committee of the Affiliated Hospital of Jiangsu University, China. Isolation and identification of BMMSCs was performed according to a previously described method (6). Briefly, bone marrow cells were diluted in an equal volume of PBS. Diluted bone marrow was isolated using 1.077 g/mL Ficoll. The isolated cells were cultured in DMEM supplemented with 10% FBS at 37°C in 5% CO_2_. Adherent cells were collected after 5 days.

Human gastric cancer cell lines (i.e., MGC-803 and HGC-27) were purchased from The Cell Bank of Type Culture Collection of Chinese Academy of Sciences (Beijing, China). HGC-27 and MGC-803 were maintained in RPMI 1640 supplemented with 10% FBS at 37°C in 5% CO_2_.

### Flow Cytometry (FCM)

To perform surface antigens staining, cells were incubated with FITC mouse anti-human CD45 (1:20; #13234199; Biolegend), FITC mouse anti-human CD19 (1:20; #4310183; ebioscience), FITC mouse anti-human CD34 (1:20; #4330486; Invitrogen), PE mouse anti-human CD90 (1:20; #4303050; ebioscience), PE mouse anti-human CD29 (1:20; #4303570; ebioscience) or PE mouse anti-human CD105 (1:20; #4300023; ebioscience) antibody at 4 °C for 30 min.

### Preparation of GCMSC Conditioned Medium (GCMSC-CM) and BMMSC Conditioned Medium (BMMSC-CM) and Co-culture With Gastric Cancer Cells

At 80% confluency, the GCMSCs or BMMSCs cells were washed with PBS and incubated with fresh DMEM supplemented with 10% FBS for 48 h. Subsequently, the supernatant was collected, filtered through a 0.22 μm filter, and diluted in a 1:1 ratio with RPMI 1640 supplemented with 10% FBS. The MGC-803 or HGC-27 cells were washed with PBS, and then treated with RPMI 1640, GCMSC-CM, GCMSC-CM+IgG (300 ng/mL), or GCMSC-CM+anti-HGF (300 ng/mL) at 37°C in 5% CO_2_ for 48 h. In the GCMSC-CM+IgG and GCMSC-CM+anti-HGF groups, IgG or anti-HGF antibodies were added to GCMSC-CM. Antibody and condition medium were incubated for 30 min at room temperature before co-culture with gastric cancer cells.

### Immunoblotting

Total proteins were extracted from the cells using the RIPA lysis buffer (Invitrogen, Carlsbad, CA, USA). After determining the protein concentration, 20 μg of the total proteins in each sample was separated using 12% sodium dodecyl sulfate polyacrylamide gel electrophoresis (SDS-PAGE), and then transferred to polyvinylidene fluoride membranes (Millipore, Billerica, MA, USA). The membranes were blocked with 5% non-fat milk and incubated with the anti-human c-Myc (dilution, 1:1,000; 13987, Cell Signaling Technology, Boston, USA), HK2 (dilution, 1:1,000; 2867, Cell Signaling Technology, Boston, USA), G6PD (dilution, 1:1,000; sc-373886, Santa Cruz Biotechnology, Texas, USA), HGF (dilution, 1:500; MAB294, R&D Systems, Minnesota, USA), PKM2 (dilution, 1:1,000; sc-365684, Santa Cruz Biotechnology, Texas, USA), p-NF-κB (dilution, 1:1,000; 3033, Cell Signaling Technology, Boston, USA), and NF-κB (dilution, 1:1,000; 8242, Cell Signaling Technology, Boston, USA) antibodies overnight at 4°C. The samples were then incubated with goat anti-mouse (dilution, 1:2,000; CW0102, CWBIO, Beijing, China) or goat anti-rabbit (dilution, 1:2,000; CW0103, CWBIO, Beijing, China) secondary antibodies for 1 h at 37°C. The blots were visualized using an enhanced chemiluminescent detection system (Amersham, UK) and analyzed employing the Image-Pro Plus version 5.1 software (Media Cybernetics, Inc., Rockville, MD, USA).

### Enzyme-Linked Immunosorbent Assay (ELISA)

The detection of HGF in BMMSC-CM and GCMSC-CM was conducted according to the MULTI SCIENCES instructions. Briefly, 300 μL of the washing buffer was added to each well for 30 s. A total of 100 μL of the standard diluted 2-fold was added to the standard well and 100 μL of the standard diluent was added to the blank well. Then, 80 μL of the assay buffer and 20 μL of the sample were added to the sample well. Subsequently, 50 μL of the diluted antibody was added to each well. The wells were covered with an adhesive strip and incubated at room temperature for 2 h using a microplate shaker set at 300 rpm. After washing six times, 100 μL of diluted streptavidin-HRP was added to each well. The wells were covered with a new adhesive strip and incubated at room temperature for 45 min using a microplate shaker set at 300 rpm. After washing six times, 100 μL of the substrate solution was added to each well and incubated for 5–30 min at room temperature. Then, 100 μL of the stop solution was added to each well and the sample was analyzed at 450 and 570 nm using a microplate reader. The calibrated optical density (OD) values were obtained by subtracting the readings obtained at 570 nm from the readings at 450 nm. A standard curve was used for the analysis.

### Immunohistochemistry

The experimental detection steps were conducted according to the instructions described in Immunohistochemistry (Boster Bio, China). After dewaxing, the paraffin section was incubated with 3% H_2_O_2_ at room temperature for 10 min. The samples were washed five times with PBS and the slices were subjected to antigen thermal repair in an antigen repair solution. The paraffin section was incubated with 5% BSA solution for 30 min at 37°C. After incubation of the anti-CD31 (dilution, 1:50; ARG52748, Arigo Biolaboratories, Shanghai, China), HK2 (dilution, 1:500; ab209847, Abcam, Cambridge, UK), c-Myc (dilution, 1:200; ab32072, Abcam, Cambridge, UK), Ki67 (dilution, 1:200; 9449, Cell Signaling Technology, Boston, USA), E-cadherin (dilution, 1:400; 3195, Cell Signaling Technology, Boston, USA), and Vimentin (dilution, 1:200; 5741, Cell Signaling Technology, Boston, USA) antibodies overnight at 4°C, the paraffin section was incubated with HRP-labeled anti-rabbit/mouse IgG for 30 min at 37°C. DAB was used for color rendering under a microscope, while hematoxylin was employed for staining. The images were collected using a microscope (Ti-S; Nikon, Tokyo, Japan).

### Transwell Migration Assay

A Transwell migration assay was used to detect the effect of GCMSCs on gastric cancer cell migration. Gastric cancer cells were co-cultured with GCMSC-CM for 48 h. The cells were collected and 6 × 10^4^ cells were seeded in the upper wells of a Transwell chamber in 200 μL of serum-free RPMI 1640. The lower compartment of the chamber was filled with 600 μL of RPMI 1640 supplemented with 10% FBS. The cells were incubated for 8 h. Following incubation, a cotton swab was used to remove the cells that did not migrate. The migrated cells were fixed with 4% formaldehyde for 30 min, stained with Crystal Violet, and photographed using a microscope. For quantitation, three random fields from each well were counted under a microscope (Ti-S; Nikon, Tokyo, Japan). Each experiment was independently repeated three times.

### Cell Proliferation Assay

Enhanced Cell Counting Kit-8 (CCK-8) (Beyotime, Shanghai, China) was used to analyze the proliferation of gastric cancer cells. A total of 5 × 10^2^ gastric cancer cells in 200 μL of DMEM were seeded in 96-well plates (Corning Inc., Corning, NY, USA) and incubated overnight at 37°C in 5% CO_2_. The plates were subsequently incubated with indicate reagent and cultured for 96 h. Then, 10 μL of CCK-8 was added to the wells, which contained 100 μL of RPMI 1640 and the plate was incubated for 1.5 h at 37°C. The optical density at 450 nm was measures utilizing a multi-well plate reader (FLx800, BioTek, Winooski, VT, USA). Each experiment was repeated three times.

### Glucose Uptake and Lactate Production Assay

Glucose uptake assays were performed to evaluate the effect of GCMSC-CM on the glucose utilization in gastric cancer cells. Briefly, 1 × 10^5^ HGC-27 or MGC-803 cells were seeded in 6-well plates (Corning Inc., Corning, NY, USA), and then treated with DMSO, GCMSC-CM. After 48 h, the cells were collected and 1 × 10^6^ cells/mL were seeded in 48-well plates (Corning Inc., Corning, NY, USA). The plated were incubated for 8 h in RPMI 1640. A clinical chemistry analyzer (Xunda, XD811, Shanghai, China) was utilized to detect the supernatant glucose concentrations using the hexokinase method. The detection of lactate in the cell culture supernatant was conducted employing a lactic acid test kit (KeyGEN BioTECH, Nanjing, China). Briefly, the sample was diluted 5-fold. Then, 20 μL of the sample was then added to 1 mL of the enzyme working fluid and 200 μL of reagent D. The reaction was mixed placed in a water bath at 37°C for 10 min. Buffer E was added and the measurement was conducted at a wavelength of 530 nm. The content of lactic acid in the sample was determined according to the following formula: The content of lactic acid = (absorbance of determination tube – absorbance of blank tube)/(absorbance of standard tube – absorbance of blank tube) × standard concentration (3 mmol/L) × dilution ratio of sample.

### Detection of Reactive Oxygen Species (ROS) in Cells

The detection of ROS in BMMSC-CM and GCMSC-CM was conducted according to the instructions of the Reactive Oxygen Species Assay Kit (Beyotime, Shanghai, China). Briefly, DCFH-DA was diluted to 10 μM in a serum-free medium. The cell culture medium was removed and DCFH-DA was added. The cells were incubated for 20 min at 37°C in a cell incubator. The cells were washed three times with serum-free cell culture medium to remove DCFH-DA. The cells were observed by fluorescence microscopy at 488 nm.

### Cell Activity Measurements

The cells treated with DMEM containing 2% serum or 200 μM H_2_O_2_ were collected. A cell suspension was prepared, which was mixed with a 0.4% Trypan Blue solution at a 9:1 ratio. Live and dead cells were counted within 3 min. Notably, the dead cells were distinctly stained blue, while the living cells were colorless and transparent. Statistical cell vitality was determined according to the following formula: Live cell rate (%) = total live cells/ (total live cells + total dead cells) × 100%.

### Gene Knockdown Using siRNA

G6PD siRNA (50 nM) (5′-TCTCAGAGGTGCAGGCCAA3′), HK2-1 siRNA (50 nM) sense (5′-CCAAGUGCAGAAGGUUGACCAGUAU-3′) antisense (5′-CCACAACUGUGAGAUUGGUCUCAUU-3′), HK2-2 siRNA (50 nM) sense (5′-GAGAAUCAGAUCUAUGCCATT-3′) antisense (5′-UGGCAUAGAUCUGAUUCUCTT-3′), and negative control (NC) (50 nM) were purchased from Guangzhou RiboBio, China. The siRNA was transfected into MGC-803, HGC-27 and GCMSCs using Lipofectamine® 2000 (Invitrogen; Thermo Fisher Scientific, Inc., USA) according to the manufacturer's instructions.

### Statistical Analysis

The data analysis was performed using the GraphPad Prism 6 software. Differences between groups were analyzed using one-way analysis of variance. The tumor growth *in vivo* was evaluated by the Kruskal-Wallis H test. A *P-*value < 0.05 was considered to be statistically significant.

## Results

### HK2 Expression Is Required for Gastric Cancer Progression and GCMSCs Promote the Expression of HK2 in Gastric Cancer Cells

**Figure 1:**
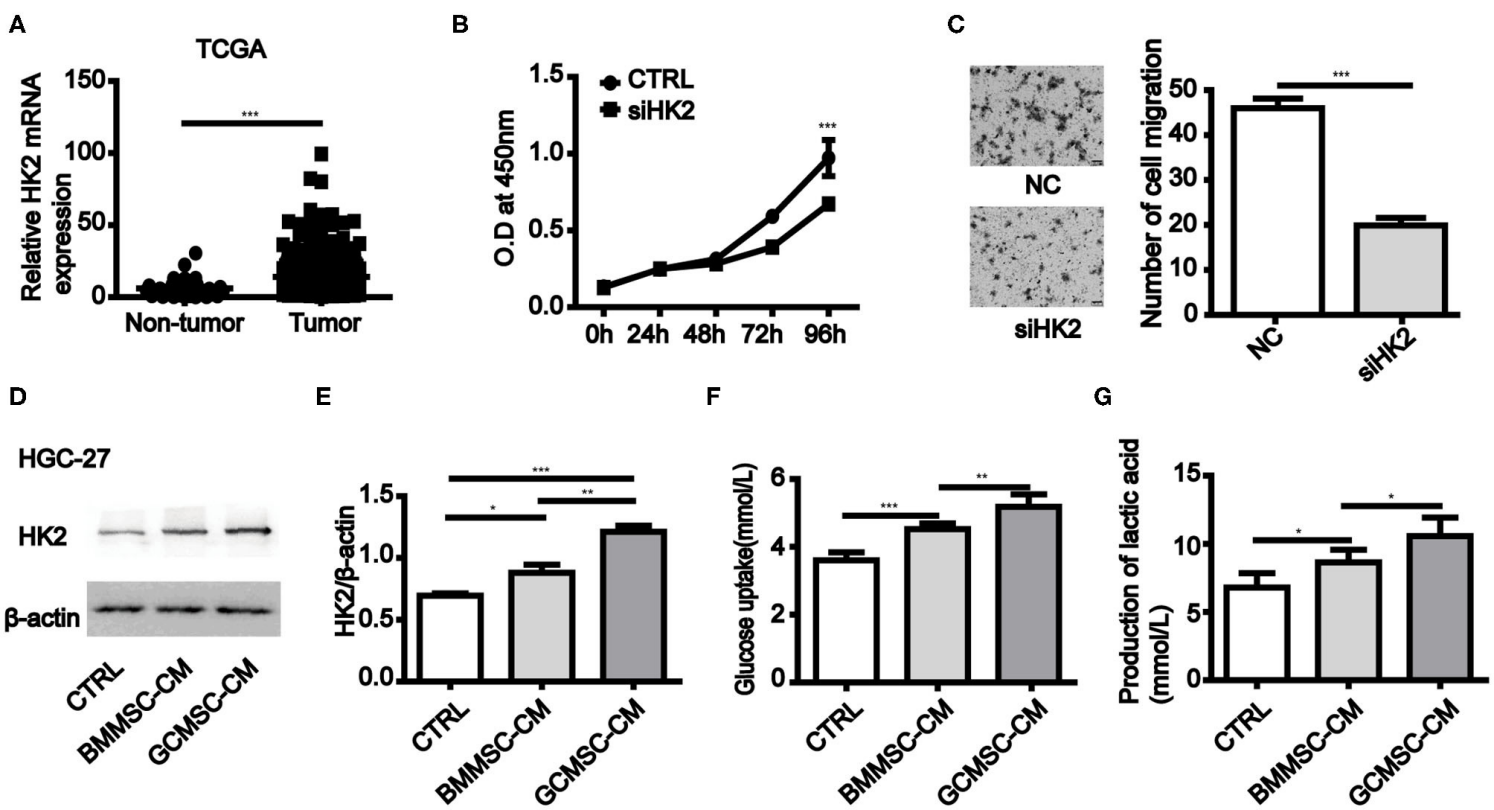
GCMSCs promote glycolysis by upregulation of HK2 in gastric cancer cells. **(A)** Expression of HK2 in non-tumor tissues and gastric cancer tissues analyzed using TCGA database (the number of non-tumor tissues was 32 and the number of gastric cancer tissues was 375). **(B)** Proliferation of HGC-27 cells transfected with siHK2 or negative control (NC) was detected by CCK-8 assays. **(C)** Migration of HGC-27 cells transfected with siHK2 or NC was detected utilizing a Transwell assay (scale bar: 50 μm). **(D)** Immunoblotting of HK2 expression in HGC-27 cells treated with BMMSC-CM or GCMSC-CM for 48h. **(E)** Quantitative statistics of HK2 expression in different groups. **(F)** Glucose uptake in HGC-27 cells treated with BMMSC-CM or GCMSC-CM for 48 h. **(G)** Lactate production in HGC-27 cells treated with BMMSC-CM or GCMSC-CM for 48 h (*n* = 3; *P < 0.05; **P < 0.01; ***P < 0.001).

To investigate the role of HK2 expression in gastric cancer cells, we first evaluated the stomach tissue data from The Cancer Genome Atlas (TCGA) database (https://www.cancer.gov/). It was found that the expression of HK2 in primary solid tumors was considerably higher than that in the normal solid tissues (Figure 1A). Inhibition of HK2 expression in HGC-27 and MGC-803 cells effectively reduced cell proliferation and migration (Figures 1B,C and Supplementary Figures 1A,B). Thus, these results indicated that HK2 played an important role in gastric cancer progression. We therefore hypothesized that GCMSCs functioned as regulators of the tumor metabolism in the tumor microenvironment. To investigate whether GCMSCs affect the metabolism on gastric cancer, GCMSCs and BMMSCs was isolated and identified. FCM analysis displayed that GCMSCs and BMMSCs were positive expression of CD90, CD29, and CD105; Nevertheless, negative expression of CD45, CD19, and CD34 (Supplementary Figures 2A,B). The expression of HK2 increased in HGC-27 and MGC-803 cells treated with BMMSC-CM or GCMSC-CM (Figures 1D,E and Supplementary Figures 1C,D). In addition, the glucose absorption and lactate production in the HGC-27 and MGC-803 cells increased following treatment with BMMSC-CM or GCMSC-CM (Figures 1F,G and Supplementary Figures 1E,F). These outcomes suggested that HK2 played an important role in the development of gastric cancer and GCMSCs could promote glycolysis in gastric cancer cells.

### GCMSCs Promotes Tumor Proliferation, Migration, and Glucose Metabolism by Upregulating HK2 *in vitro*

**Figure 2:**
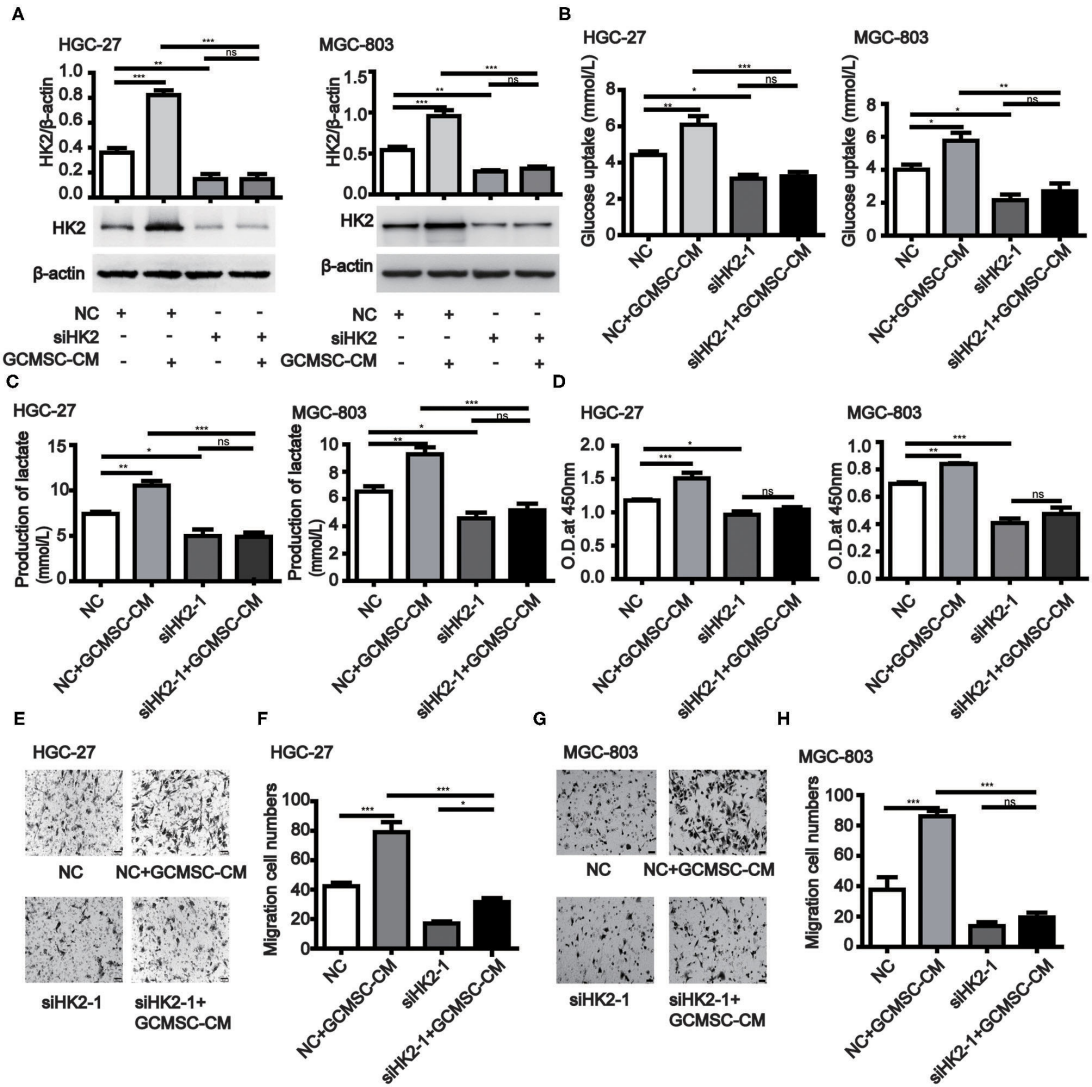
GCMSCs facilitate glucose metabolism, proliferation, and migration of gastric cancer cells by regulating HK2. **(A)** Immunoblotting of the HK2 expression in HGC-27 and MGC-803 cells treated with the indicated reagent following transfection with siHK2-1 or NC (down). Quantitative statistics of the HK2 expression in different groups (up). **(B)** Glucose uptake in HGC-27 and MGC-803 cells treated with the indicated reagent following transfection with siHK2-1 or NC. **(C)** Lactate production in HGC-27 and MGC-803 cells treated with the indicated reagent following transfection with siHK2-1 or NC. **(D)** Proliferation of HGC-27 and MGC-803 cells treated with the indicated reagent following transfection with siHK2-1 or NC detected by CCK-8. **(E)** Migration of HGC-27 cells treated with the indicated reagent following transfection with siHK2-1 or NC detected using the Transwell assay. **(F)** Quantitative statistics of the HGC-27 cell migration in different groups. **(G)** Migration of MGC-803 cells treated with the indicated reagent following transfection with siHK2-1 or NC detected using the Transwell assay. **(H)** Quantitative statistics of the MGC-803 cell migration in different groups. (*n* = 3; *P < 0.05; **P < 0.01; ***P < 0.001).

To further investigate the effect of HK2 induced by GCMSCs on gastric cancer cell proliferation, migration, and glucose metabolism, we analyzed the tumor cell processes. The expression of HK2 was decreased in HK2 deficient HGC-27 and MGC-803 cells (Figure 2A and Supplementary Figures 3A,B). HK2 deficiency also effectively inhibited the promoting effect of GCMSCs on the glucose uptake and lactate production of the tumor cells (Figures 2B,C and Supplementary Figures 3C,D). The results of the HK2 deficiency study demonstrated that HK2 played an important role in tumor cell proliferation promoted by GCMSCs (Figure 2D and Supplementary Figure 3E). Furthermore, inhibiting the expression of HK2 in HGC-27 and MGC-803 cells decreased tumor migration induced by GCMSCs (Figures 2E–H and Supplementary Figures 3F,G). These outcomes indicated that GCMSCs could regulate the proliferation, migration, and metabolism of gastric cancer cells by upregulating HK2 *in vitro*.

### GCMSCs-Derived HGF Promotes HK2 Expression in Gastric Cancer Cells

**Figure 3:**
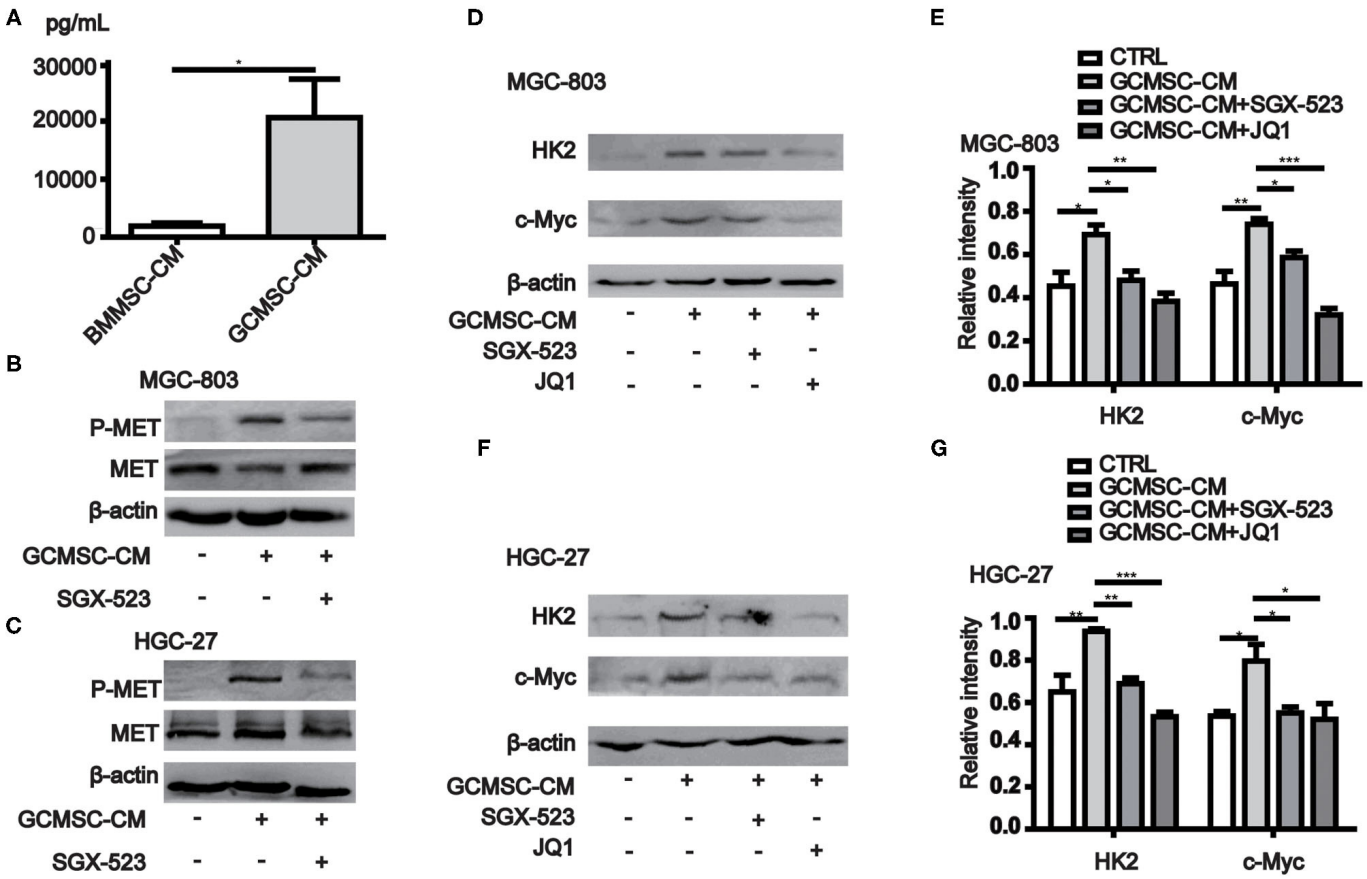
GCMSCs-derived HGF promotes HK2 expression by regulating c-Myc. **(A)** ELISA analysis of the HGF expression in BMMSC-CM and GCMSC-CM. **(B)** Immunoblotting of p-MET, total MET in MGC-803 cells treated with GCMSC-CM or GCMSC-CM+SGX-523 (4 nM) for 30 min. **(C)** Immunoblotting of p-MET, total MET in HGC-27 cells treated with GCMSC-CM or GCMSC-CM+SGX-523 (4 nM) for 30 min. **(D)** Immunoblotting of HK2 and c-Myc in MGC-803 cells treated with GCMSC-CM, GCMSC-CM+SGX-523 (4 nM), or GCMSC-CM+JQ1 (0.8 μM) for 48 h. **(E)** Quantitative statistics of the c-Myc and HK2 expression in MGC-803 cells. **(F)** Immunoblotting of HK2 and c-Myc in HGC-27 cells treated with GCMSC-CM, GCMSC-CM+SGX-523 (4 nM), or GCMSC-CM+JQ1 (0.8 μM) for 48 h. **(G)** Quantitative statistics of the c-Myc and HK2 expression in HGC-27 cells (*n* = 3; *P < 0.05; **P < 0.01; ***P < 0.001).

Previous studies have shown that the expression of HK2 in tumor cells is regulated by c-Myc. Moreover, the data obtained from the cBioPortal database (http://www.cbioportal.org/) revealed that the expression of MYC in gastric cancer cells correlated with the expression of MET (Table 1) (28, 29). The conducted ELISA analysis confirmed that the expression of HGF in GCMSCs was higher than that in BMMSCs (Figure 3A). Hence, we hypothesized that GCMSCs could upregulate the expression of HK2 in gastric cancer cells by secreting HGF. In addition, it was determined that SGX-523, a MET phosphorylation inhibitor, could effectively inhibit the phosphorylation of MET signaling in tumor cells (Figures 3B,C). Inhibiting the HGF signal in the HGC-27 and MGC-803 cells resulted in a decrease in the upregulation of the HK2 and c-Myc expression by GCMSCs. Concurrently, JQ1 inhibited the expression of c-Myc in tumor cells. On the other hand, GCMSCs failed to upregulate the expression of HK2 in the MGC-803 (Figures 3D,E) and HGC-27 cells (Figures 3F,G) following treatment with JQ1. These results implied that HGF derived from GCMSCs promoted the HK2 expression by upregulating c-Myc in gastric cancer cells.

**Table 1:** The trendency of MET, HK2, and MYC expression in gastric cancer tissues.

**A**	**B**	**Neither**	**A Not B**	**B Not A**	**Both**	***p*-value**	**Tendency**
MET	MYC	347	25	30	10	<0.001	Co-occurrence
MET	HK2	362	31	15	4	0.067	Co-occurrence

*cBioPortal for cancer genomics was used to analysis the trendency of MET, HK2, and MYC in gastric cancer tissues*.

### The Expression of G6PD Maintains the Survival of MSCs and Promotes the Production of HGF in MSCs

**Figure 4:**
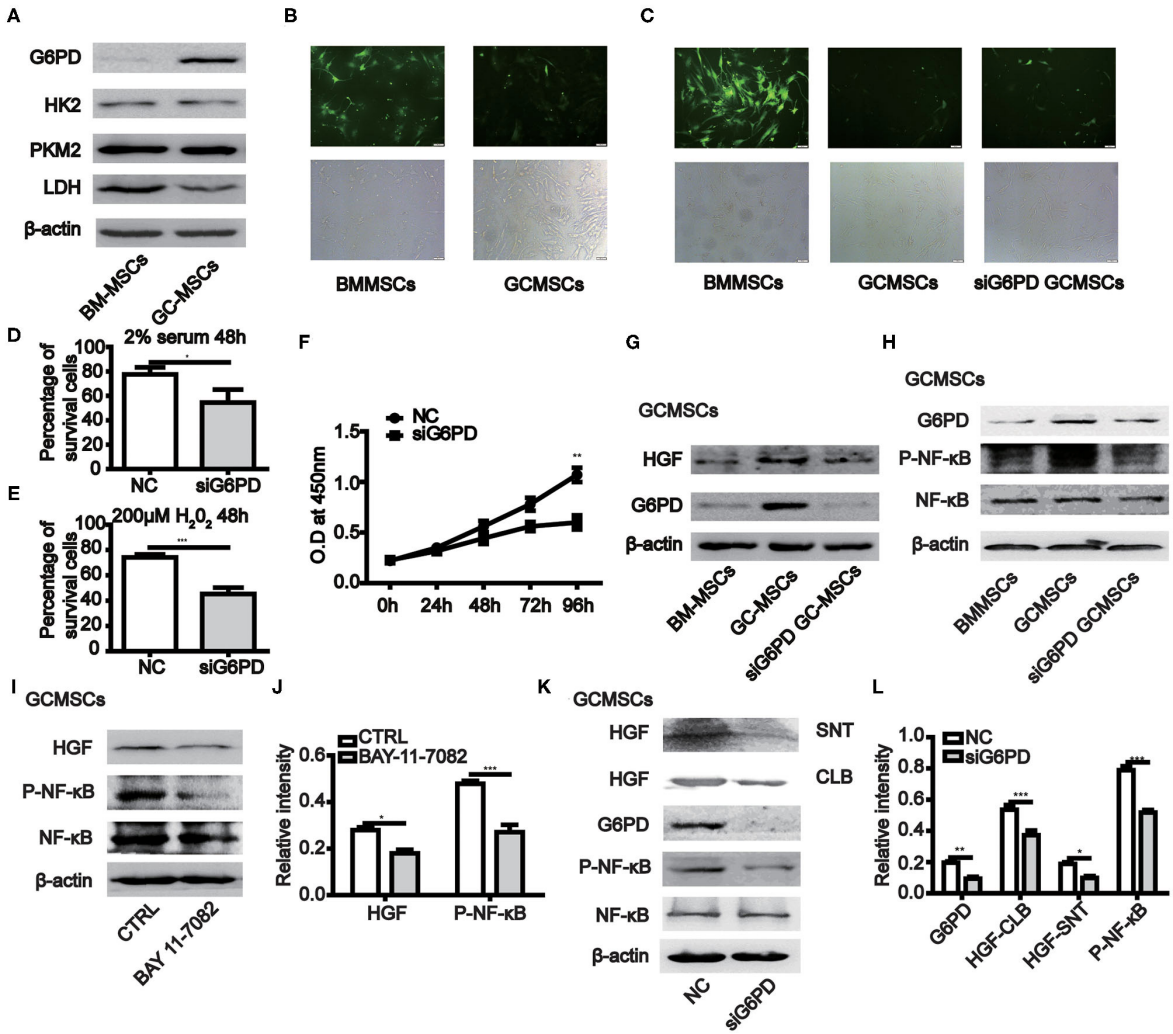
The expression of G6PD in MSCs promotes the growth of MSCs and the production of HGF. **(A)** Immunoblotting of the HK2, PKM2, LDH, and G6PD expression in BMMSCs and GCMSCs. **(B)** ROS levels in BMMSCs and GCMSCs were detected using the ROS assay kit. **(C)** ROS level in BMMSCs, GCMSCs, and GCMSCs transfected with siG6PD was detected using the ROS assay kit. **(D,E)** Cell viability assay of GCMSCs treated with DMEM containing 2% serum **(D)** or 200 μM H_2_O_2_ **(E)** following transfection with siG6PD or NC for 48 h. **(F)** Proliferation of GCMSCs transfected with siG6PD or negative control (NC) was detected by CCK-8 assays. **(G)** Immunoblotting of the G6PD and HGF expression in BMMSCs, GCMSCs, and GCMSCs transfected with siG6PD. **(H)** Immunoblotting of p-NF-κB, total NF-κB, and G6PD in BMMSCS, GCMSCs, and GCMSCs transfected with siG6PD. **(I)** Immunoblotting of p-NF-κB, total NF-κB, and HGF in GCMSCs treated with BAY 11-7082 (5 nM) and untreated GCMSCs. **(J)** Quantitative statistics of the p-NF-κB and HGF expression in different groups. **(K)** Immunoblotting of p-NF-κB, total NF-κB, supernatant (SNT) HGF, and HGF in GCMSCs (CLB) transfected with siG6PD and GCMSCs not transfected with siG6PD. **(L)** Quantitative statistics of the G6PD, p-NF-κB, and HGF expression in different groups (*n* = 3; *P < 0.05; **P < 0.01; ***P < 0.001).

When BMMSCs and GCMSCs were cultured *in vitro*, the growth state of the latter was comparatively better. Hence, we detected the key enzymes participating in the glycolysis and pentose phosphate pathways (PPP) in both kinds of MSCs. The results demonstrated that the expression of G6PD, a key enzyme in PPP, was higher in GCMSCs than in BMMSCs. However, the expression of the key glycolytic enzymes was comparable in both types of MSCs (Figure 4A). The PPP has the function of scavenging intracellular ROS. Thus, we used a ROS fluorescence probe to detect such species in BMMSCs and GCMSCs (Figure 4B). It was found that BMMSCs exhibited higher ROS levels than GCMSCs. After inhibiting the expression of G6PD in GCMSCs, the level of ROS in the cells increased (Figure 4C). Furthermore, serum deprivation or H_2_O_2_ were used to detect the resistance of GCMSCs with different G6PD levels to extracellular adverse stimulation. Inhibition of G6PD in GCMSCs led to decreased resistance to extracellular adverse stimuli (Figures 4D,E). To further investigate whether G6PD affects GCMSCs proliferation, we performed a cell proliferation assay in G6PD-silenced GCMSCs. Inhibition of G6PD expression in GCMSCs effectively reduced cell proliferation (Figure 4F). In addition, protein microarray results revealed that BMMSCs and GCMSCs differed in cytokine production, particularly HGF. It was determined that the expression of G6PD regulate the production of HGF in GCMSCs (Figure 4G). We then confirmed that the activation of the NF-κB signaling could be regulated by G6PD (Figure 4H). Moreover, NF-κB signaling regulated the HGF production in GCMSCs (Figures 4I,J). Subsequently, the production of HGF in G6PD-knockdown GCMSCs was assessed. It was found that a reduction in the G6PD expression resulted in a significant decrease in the production of HGF (Figures 4K,L). These results suggested that G6PD promoted the production of HGF by regulating the NF-κB signaling in GCMSCs.

### Blocking GCMSCs-Derived HGF Decreased Tumor Proliferation and Metastasis *in vivo*

**Figure 5:**
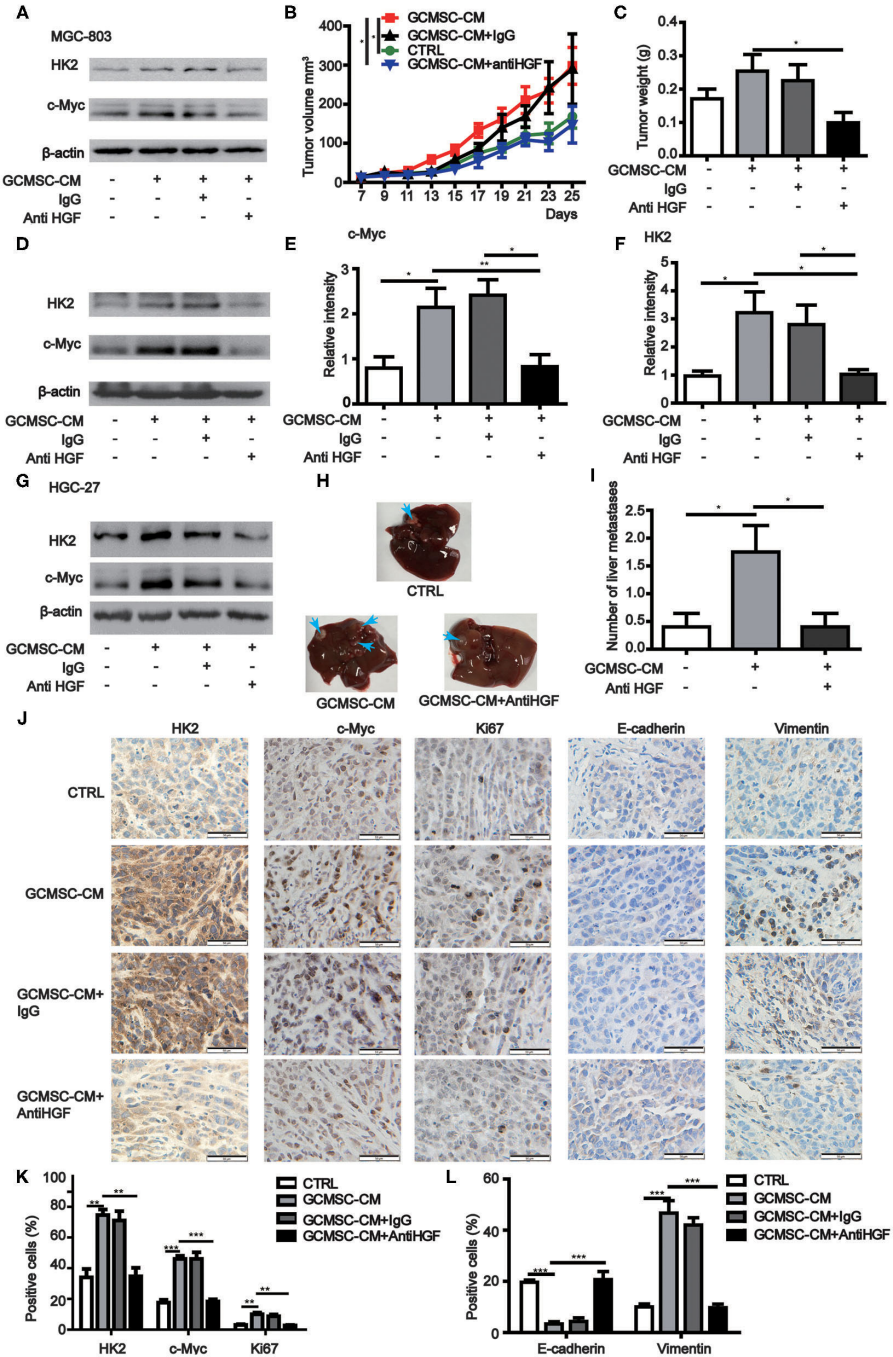
GCMSCs-derived HGF promotes proliferation, metastasis, and angiogenesis of gastric cancer cells *in vivo*. **(A)** Immunoblotting of the HK2 and c-Myc expression in MGC-803 cells treated with the indicated reagent for 48 h *in vitro*. **(B,C)** Growth curves **(B)** and tumor weights at day 28 **(C)** of mice injected subcutaneously with MGC-803 cells treated with the indicated reagent. Volumes of visible tumors were measured every 2 days (*n* = 5). **(D)** Immunoblotting of the c-Myc and HK2 expression in tumor tissues. **(E)** Quantitative statistics of the c-Myc expression in different groups of tumor tissues. **(F)** Quantitative statistics of the HK2 expression in different groups of tumor tissues. **(G)** Immunoblotting of the HK2 and c-Myc expression in HGC-27 cells treated with the indicated reagent for 48 h *in vitro*. **(H)** Images of the liver from mice intraperitoneally injected with HGC-27 cells treated with the indicated reagent. **(I)** Quantitative statistics of liver metastases in different groups. **(J)** Immunohistochemistry of c-Myc, HK2, Ki67, E-cadherin, and Vimentin in mice tumor tissues (scale bar: 50 μm). **(K,L)** Quantification of **(J)** (*P < 0.05; **P < 0.01; ***P < 0.001).

To further confirm that GCMSCs-derived HGF promoted tumor progression by upregulating HK2 in gastric cancer cells, we constructed a mouse tumor model. It was determined that a HGF neutralizing antibody could effectively inhibit the upregulation of HK2 expression by GCMSCs-derived HGF *in vitro* (Figure 5A). GCMSCs effectively promoted tumor proliferation. Conversely, inhibition of GCMSCs-derived HGF signaling reduced the promoting function *in vivo* (Figures 5B,C). Concurrently, we also assessed the expression of HK2 and c-Myc in the tumor tissues using western blots (Figures 5D–F) or immunohistochemical analysis (Figures 5J,K). The expression of HK2 and c-Myc increased following GCMSC-CM treatment. Moreover, when HGF signaling derived from GCMSCs was blocked, the expression of HK2 and c-Myc decreased. Hence, GCMSCs-derived HGF promoted tumor proliferation by upregulating c-Myc-HK2 *in vivo*. To confirm that GCMSCs-derived HGF could regulate tumor metastasis by upregulating HK2, we first investigated whether a HGF neutralizing antibody could inhibit the upregulation of HK2 expression by GCMSCs-derived HGF in HGC-27 cells *in vitro* (Figure 5G). Subsequently, a tumor model was constructed in mouse peritoneal cavity. The results implied that GCMSCs-derived HGF promoted abdominal tumor metastasis to the liver and inhibition of HGF signaling effectively reduced the promoting effects of GCMSCs *in vivo* (Figures 5H,I). The immunohistochemical analysis outcomes demonstrated that HGF derived from GCMSCs upregulated Vimentin and downregulated E-cadherin in gastric cancer tissues (Figures 5J,L). Previous studies showed that HGF plays a role in promoting tumor angiogenesis. In addition, it has been reported that endothelial growth can be promoted by upregulating the expression of c-Myc and HK2. Accordingly, the immunohistochemical evaluation revealed that HGF derived from GCMSCs promoted tumor angiogenesis (Supplementary Figures 4A,B). Overall, the data obtained herein indicated that GCMSCs-derived HGF promoted gastric cancer progression by upregulating HK2 *in vivo*.

## Discussion

In a tumor microenvironment, cells have the ability to reprogram chemotactic non-tumor cells. Reprogramming of lipid metabolism in cancer-associated fibroblasts (CAF) promotes the migration of colorectal cancer cells. Furthermore, increased expression of fatty acid synthase (FASN) in CAFs promotes lipid metabolism. It has been shown that colorectal cancer cells exhibit increased migration by taking up lipid metabolites secreted by CAFs (30). In addition to regulating glucose metabolism, G6PD also controls cell signal transduction. Moreover, the enzyme regulates the intracellular ROS content by controlling NADPH production. It has been shown that the activity of STAT3 is also affected by G6PD and NADPH (31). On the other hand, the activation and inhibition of NF-κB signaling is regulated by H_2_O_2_ and depends on the cytosolic and nuclear content of this species (32). In the present study, we found that the production of HGF by GCMSCs increased as a result of G6PD-NF-κB signaling. Previous investigations demonstrated that the expression of G6PD in tumor cells contributed to resistance to various chemotherapeutic agents, including oxaliplatin (13). However, regulation of chemotherapy resistance by G6PD expression in GCMSCs requires further assessment.

The majority of cancer cells alter their metabolism via metabolic reprogramming (33). Increased glycolysis is a particularly important feature of the metabolic reprogramming in cancer cells (34). HGF secreted in a paracrine manner by tumor microenvironment-localized stroma cell and not by the tumor cells themselves has been shown to promote tumor proliferation, metastasis, and therapeutic resistance (35, 36). On the other hand, blocking the HGF/c-MET signaling induces cell cycle arrest, DNA damage, and apoptosis (37). It is known that HGF facilitates metabolic reprogramming by regulating the Warburg effect and glutaminolysis in liver cancer cells (38). In a NSCLC model, inhibition of Met resulted in downregulation of HK2, which is important for the initiation of glycolysis (36). Our results demonstrated GCMSCs-derived HGF reprogramed glucose metabolism of gastric cancer cells by regulating HK2. GCMSCs produce a variety of cytokines, which can upregulate c-Myc. Hence, blocking GCMSCs-derived HGF alone cannot completely reduce the upregulation of HK2-c-Myc. Inhibition of the HK2 expression could effectively inhibit the glucose uptake, lactate production, proliferation, and migration of gastric cancer cell lines. In addition, blocking GCMSCs-derived HGF could decrease tumor proliferation and metastasis *in vivo*. Injecting tumor cells through the tail vein is the classic method used to detect tumor metastasis. The metabolic reprogramming of tumor cells induced by GCMSCs requires continuous stimulation (26). Hence, tumor cells injected intraperitoneally were selected to provide sustained cell signaling from GCMSCs.

Inhibition of HK2 expression in tumor cells effectively depressed the development of tumor cells. These results were previously confirmed in several experimental studies (39, 40). However, when analyzing the survival curve (the data from Kaplan Meier-plotter) for gastric cancer patients, it was found that the patients exhibiting high HK2 expression had a longer survival time than those with low HK2 expression. When analyzing the survival curve (the data from TCGA) for gastric cancer patients, it was found that the expression of HK2 in tumor tissues had no effect on survival (Supplementary Figures 5A,B). Moreover, among patients receiving 5-FU treatment, the survival rate was lower when the expression of HK2 was high (Supplementary Figure 5C). Conversely, the survival rate was higher when the HK2 expression was low. The results of the clinical studies and basic experimental investigations were inconsistent; therefore, further evaluation is needed to elucidate the differences. Even though many targeted drugs for the treatment of gastric cancer have been developed, 5-FU remains an important chemotherapeutic agent. By analyzing patient survival curves, the expression of HK2 in cancer tissues may become an indicator for 5-FU treatment in gastric cancer chemotherapy. The effects of tumor stromal cells on 5-FU in the treatment of gastric cancer remain unexplored. Tumor stromal cells play an important role in the occurrence and development of tumors. In addition, in the process of tumor therapy, clinicians should not always focus on finding the target of the tumor itself. Importantly, inhibiting of tumor development by blocking GCMSCs-derived HGF may become a new target for the treatment of gastric cancer.

## Conclusion

In conclusion, our findings demonstrate that the expression of G6PD activated NF-κB signaling and stimulated the production of HGF in GCMSCs. HGF derived from GCMSCs promoted glycolysis, proliferation, and metastasis of gastric cancer by upregulating c-Myc-HK2 signal. Blocking GCMSCs-derived HGF decreased the progression of gastric cancer *in vivo*.

## Data Availability Statement

The raw data supporting the conclusions of this article will be made available by the authors, without undue reservation.

## Ethics Statement

The animal study was reviewed and approved by the local ethics committee of the Jiangsu University, China.

## Author Contributions

BC, CH, and TC participated in the experimental design, manuscript writing, and performed the experiments. YZ, SG, QW, and LS were involved in establishing the animal models. ZC and ZH contributed to the collection of gastric cancer tissues and bone marrow. MW, WX, XZ, and RX participated in the correction of the manuscript. WZ and BS contributed to the experimental design, manuscript writing, manuscript revision, and data analysis. All authors contributed to the article and approved the submitted version.

## Conflict of Interest

The authors declare that the research was conducted in the absence of any commercial or financial relationships that could be construed as a potential conflict of interest.
