# Supplementary material for: G6PD-NF-κB-HGF Signal in Gastric Cancer-Associated Mesenchymal Stem Cells Promotes the Proliferation and Metastasis of Gastric Cancer Cells by Upregulating the Expression of HK2
Source: Front Oncol. 2021 Feb 26;11:648706. doi: 10.3389/fonc.2021.648706 (PMC7952978; doi:10.3389/fonc.2021.648706)
Supplement: Supplementary file 1 [file Data_Sheet_1.docx]

**Supplementary information**


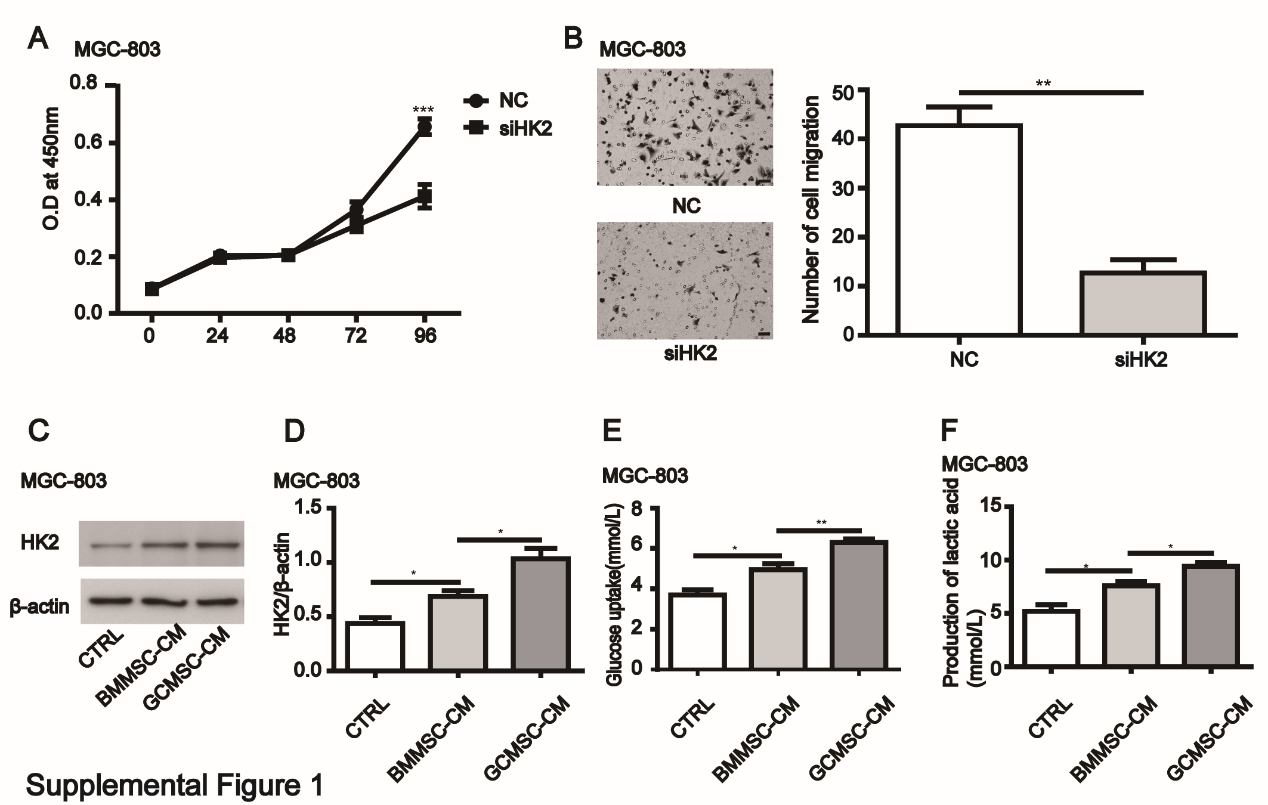


**Fig. S1** GCMSCs promote glycolysis by upregulation of HK2 in gastric cancer cells. **(A)** Proliferation of MGC-803 cells transfected with siHK2 or negative control (NC) was detected by CCK-8 assays. **(B)** Migration of MGC-803 cells transfected with siHK2 or NC was detected utilizing a Transwell assay (scale bar: 50 μm). **(C)** Immunoblotting of HK2 expression in MGC-803 cells treated with BMMSC-CM or GCMSC-CM for 48h. **(D)** Quantitative statistics of HK2 expression in different groups. **(E)** Glucose uptake in MGC-803 cells treated with BMMSC-CM or GCMSC-CM for 48 h. **(F)** Lactate production in MGC-803 cells treated with BMMSC-CM or GCMSC-CM for 48 h. (n = 3; ^*^*P* < 0.05; ^**^*P* < 0.01; ^***^*P* < 0.001).


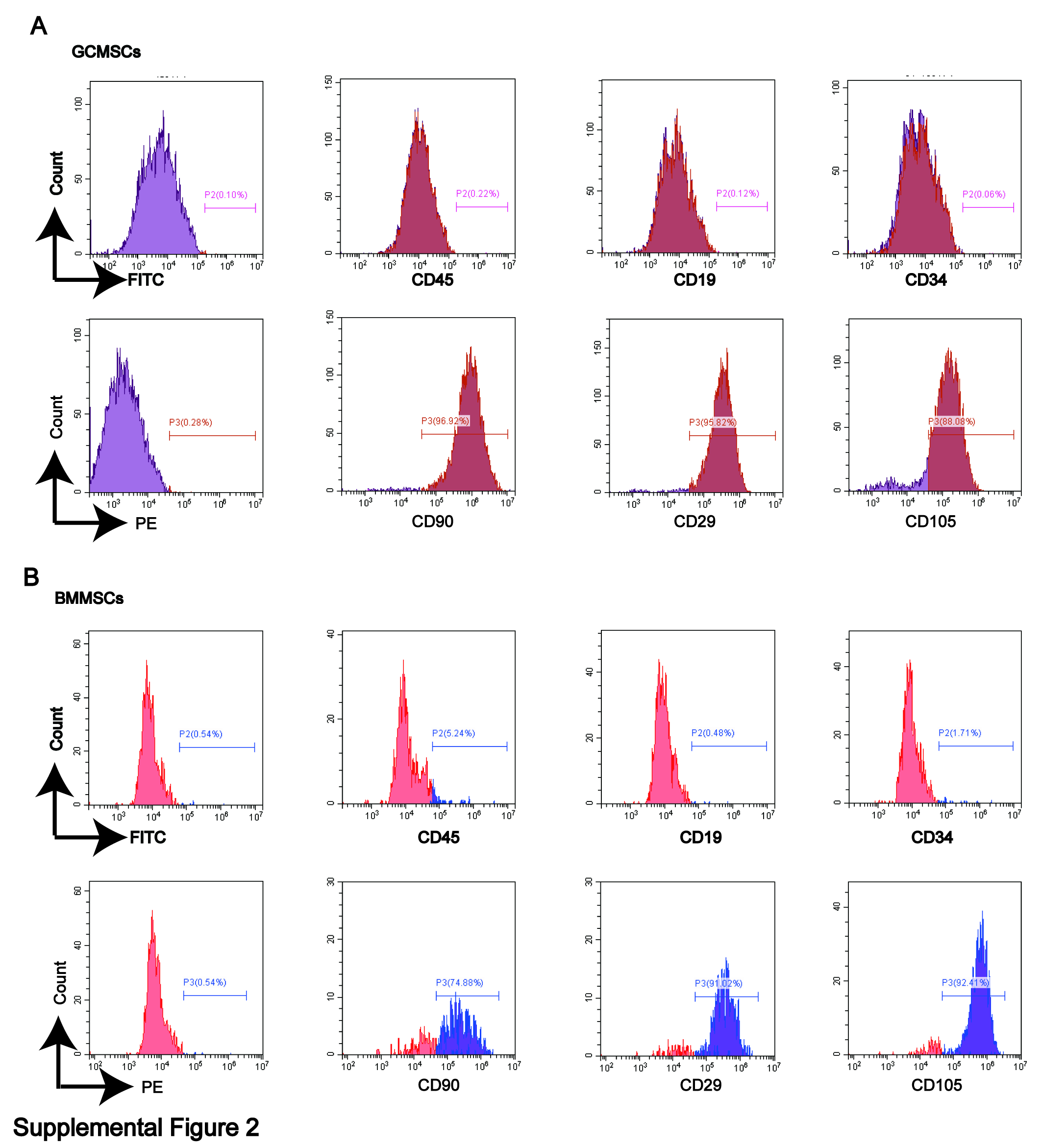


**Fig. S2** Identification of mesenchymal stem cells. (A) Human CD45, CD19, CD34, CD90, CD29 and CD105 in GCMSCs were detected by FCM. (B) Human CD45, CD19, CD34, CD90, CD29 and CD105 in BMMSCs were detected by FCM.


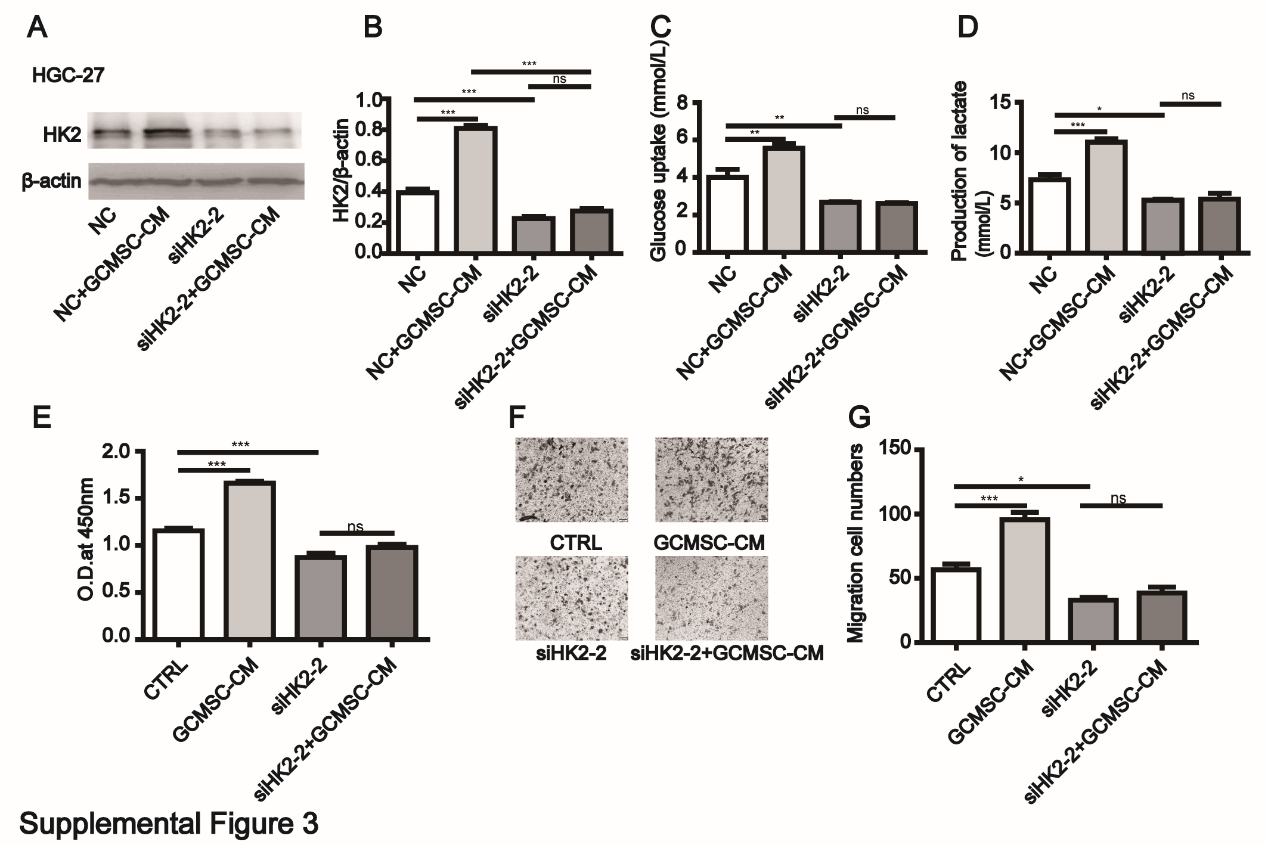


**Fig. S3** GCMSCs facilitate glucose metabolism, proliferation and migration of gastric cancer cells via regulating HK2. (**A)** Immunoblotting of the HK2 expression in HGC-27 cells treated with the indicated reagent after transfected with siHK2-2 or NC. **(B)** Quantitative statistics of the HK2 expression in different groups **(C)** Glucose uptake in HGC-27 cells treated with the indicated reagent after transfected with siHK2-2 or NC. **(D)** Lactate production in HGC-27 cells treated with the indicated reagent after transfected with siHK2-2 or NC. **(E)** Proliferation of HGC-27 cells treated with the indicated reagent after transfected with siHK2-2 or NC was detected by CCK-8. **(F)** Migration of HGC-27 cells treated with the indicated reagent after transfected with siHK2-2 or NC was detected by transwell assay. **(G)** Quantitative statistics of the HGC-27 cell migration in different groups (n=3; ^*^*P*<0.05; ^**^*P*<0.01; ^***^*P*<0.001).


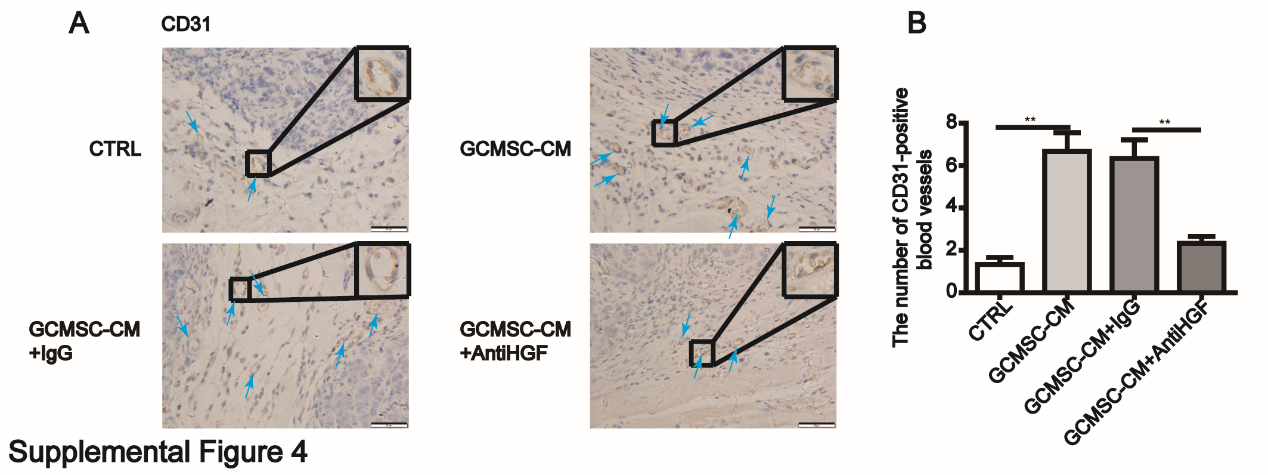


**Fig. S4** GCMSCs derived HGF promotes tumor angiogenesis.

**(A)** Immunohistochemistry of CD31 in tumor tissue of mice. **(B)** Quantitative statistics of the number of CD31 positive blood vessels under high power mirror in different groups of tumor tissues. (^**^*P*<0.01).


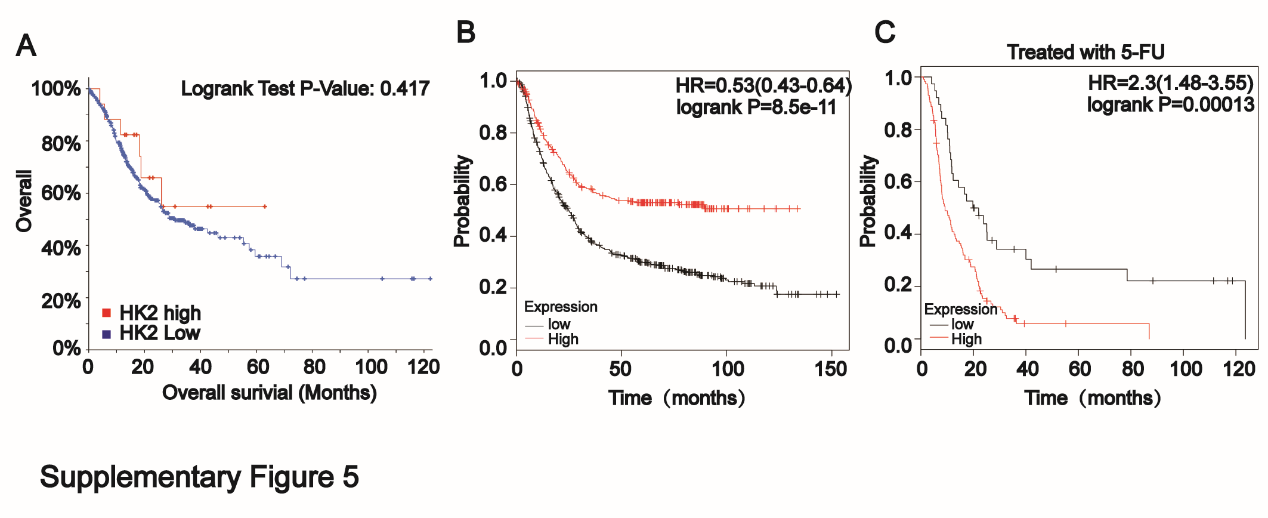


**Fig. S5 Effect of HK2 expression on overall survival of patients with gastric cancer.** **(A)** Effect of HK2 expression on overall survival of gastric cancer patients (the data from TCGA). **(B)** Effect of HK2 expression on overall survival of gastric cancer patients (the data from Kaplan Meier-plotter. 202934_at). **(C)** Effect of HK2 expression on overall survival of gastric cancer patients with 5-FU therapy (the data from Kaplan Meier-plotter. 202934_at).
